# Association between *C1q* gene polymorphisms and autoimmune thyroid diseases

**DOI:** 10.1590/2359-3997000000256

**Published:** 2017-02-01

**Authors:** Qiuming Yao, Jie Li, Xiaofei An, Wenjuan Jiang, Qiu Qin, Ronghua Song, Ni Yan, Danfeng Li, Yanfei Jiang, Wen Wang, Liangfeng Shi, Jin-an Zhang

**Affiliations:** 1 Department of Endocrinology Jinshan Hospital Fudan University Shanghai China Department of Endocrinology, Jinshan Hospital of Fudan University, Shanghai, China; 2 Department of Nephrology Xi’an Central Hospital Xi’an China Department of Nephrology, Xi’an Central Hospital, Xi’an, China

**Keywords:** Autoimmune thyroid diseases, C1q, single nucleotide polymorphism

## Abstract

**Objective:**

In the present study, we aimed to assess the associations of C1q gene polymorphisms with autoimmune thyroid diseases (AITD) susceptibility.

**Subjects and methods:**

A set of 1,003 AITD patients (661 with Graves’ disease and 342 with Hashimoto’s thyroiditis) and 880 ethnically- and geographically-matched controls from Chinese Han population were included. Five common single nucleotide polymorphisms (SNPs) (rs294185, rs292001, rs682658, rs665691 and rs294179) in C1q gene locus were genotyped. Frequencies of genotypes and alleles were compared between patients and controls, and haplotype analysis was also performed.

**Results:**

There was no statistically significant difference between AITD patients and controls in the frequencies of alleles of rs294185 (P = 0.41), rs292001 (P = 0.71), rs682658 (P = 0.68), rs665691 (P = 0.68) and rs294179 (P = 0.69). There was also no statistically significant difference between AITD patients and controls in the frequencies of genotypes of rs294185 (P = 0.72), rs292001 (P = 0.89), rs682658 (P = 0.83), rs665691 (P = 0.90) and rs294179 (P = 0.43). Stratified analyses showed that none of those five SNPs in C1q gene were associated with Graves’ disease or Hashimoto’s thyroiditis (all P values > 0.05). Haplotype analysis revealed that there were no obvious genetic associations of C1q gene polymorphisms with AITD susceptibility.

**Conclusions:**

We, for the first time, identified the associations between C1q gene SNPs and AITD, and our findings suggested that five common SNPs in C1q gene were not associated with AITD susceptibility in Chinese Han population.

## INTRODUCTION

Autoimmune thyroid diseases (AITD) are common autoimmune disorders in endocrinologic system, affecting about 5% of overall population (1). AITD is characterized by immune imbalance and auto-antibodies towards thyroid. As the most common type of AITD, Hashimoto’s thyroiditis (HT) mainly causes hypothyroidism, and it is characterized by lymphocytic infiltration and presence of thyroid peroxidase antibodies (TPOAb) or thyroglobulin antibody (TgAb). Graves’ disease (GD) is another main type of AITD, which is characterized by hyperthyroidism due to overproduction of thyroid hormones induced by specific auto-antibodies against thyrotropin receptor (TSHR).

It has been known that many factors are involved in the initiation and development of AITD, such as genetic factors, environmental factors and nutritional elements, such as iodine intake and infection (2,3). However, the pathogenesis of AITD remains unclear. In the past decade, several genetic polymorphisms have been found to be associated with AITD susceptibility, such as genetic polymorphisms in the genes encoding TSHR, human leukocyte antigen (*HLA*) and cytotoxic T lymphocyte-associated antigen-4 (*CTLA4*) (4-7). Other genetic polymorphisms associated with AITD susceptibility have also been reported, such as polymorphisms in the *CD40*, *IL-17*, *FCRL3* and protein tyrosine phosphatase-22 (PTPN22) genes (8-11). However, the above-mentioned genetic polymorphisms can only explain part of gene susceptibility to AITD, and other genetic polymorphisms are believed to have important roles in AITD, which need to be explored in future studies.

Complement is a vital part of innate immune system in human body, which can be activated by three different pathways (12). The classical pathway of complement activation is characterized by the binding of C1q to immune complexes (13). *C1q* is a recognition component in the classical pathway, and it can help solubilize immune complexes and aid in the clearance of apoptotic debris (14). The gene coding region for *C1q* is localized on chromosome 1p34–36 and consists of three genes, *C1qA*, *C1qB* and *C1qC* (15). Several single nucleotide polymorphisms (SNPs) have been found in the *C1q* gene, such as rs292001, rs682658, rs665691 and rs294179. SNPs in the *C1q* gene have been reported to be associated with several common autoimmune diseases, such as systemic lupus erythematosus (SLE) and rheumatoid arthritis. However, the roles of *C1q* gene SNPs in AITD susceptibility remain poorly explored. In the present study, the associations of five common SNPs in *C1q* gene with AITD susceptibility were examined in order to identify additional risk variants for AITD susceptibility. We, for the first time, clarified the roles of *C1q* gene SNPs in AITD susceptibility in Chinese Han population.

## SUBJECTS AND METHODS

### Subjects

The present study was a case-control study, and all subjects were from Chinese Han population. A set of 1,003 AITD patients (661 with GD and 342 with HT) and 880 ethnically- and geographically-matched controls were included. All controls were healthy and unrelated to AITD patients. The AITD patients were recruited in the outpatient department of Jinshan Hospital of Fudan University (Shanghai, China). AITD patients were diagnosed according to clinical guidelines, which were described in details in our previously published studies (16). The diagnosis of GD was based on the presence of clinical and laboratory biochemical hyperthyroidism with diffuse goiter, decreased TSH value as well as increased levels of free thyroid hormones and anti-thyroid stimulating hormone receptor antibody (TRAb) (+). HT was diagnosed by the presence of an enlarged thyroid and TPOAb (+) or TgAb (+). The healthy subjects without thyroid goiters, autoimmune diseases and family history were randomly recruited from health examination center of Jinshan Hospital of Fudan University (Shanghai, China) and used as controls. All the controls presented no TRAb(+), TPOAb(+) or TgAb(+). The research was approved by the Ethics Committee of Jinshan Hospital of Fudan University (Shanghai, China), and informed consents were obtained from all included participants.

### Genotyping

About 2 mL peripheral venous blood was collected from patients and controls. Genomic DNA was extracted from the collected blood samples, and the concentration and purity of DNA samples were determined by Nano Drop 2000 Spectro-photometer (Thermo, USA). From Hapmap CHB database, five SNPs of *C1q* gene (rs292001/rs682658/rs294185/rs665691/rs294179) were selected according to the predisigned eligibility criteria as follows: (1) the frequency of minor allele was greater than 0.10; and (2) P value for Hardy-Weinberg equilibrium (HWE) was greater than 0.001. The genotyping of five SNPs of *C1q* was conducted by using ligase detection reaction (LDR) platform (16). The target DNA sequences of those five SNPs of *C1q* gene were amplified using multiplex polymerase chain reaction (PCR) method. The sequences of primers for those five SNPs were as follows:

(1) rs294185:

Forward: 5’-ACCCCAGCTTTGACATTTGC-3’;

Reverse: 5’-GGTGTGGTCTCAGTTTTAGG-3’;

(2) rs292001:

Forward: 5’-TCCTAGTCCAAAGCAGACCA-3’;

Reverse: 5’-GTTCAGGTACCACATGTAGG-3’;

(3) rs665691:

Forward: 5’-AAGCATTCTCAGGGTCCAAG-3’;

Reverse: 5’-CCTTAACTGATGGGATGCTC-3’;

(4) rs294179:

Forward: 5’-GCACATCTTGCCTTTGTCTG-3’;

Reverse: 5’-CCTGTGCTGAACTTCAGGAG-3’;

(5) rs682658:

Forward: 5’-ACTTGGCCCTAGGAGTCCCT-3’;

Reverse: 5’-CAGCCCCATAATGCAGTATC-3’.

### Statistical analysis

Statistical analyses were carried out by using SPSS (version 17.0). Chi-square test was used to detect the difference in the frequencies of genotypes and alleles between patients and controls. The association between SNPs and AITD susceptibility was firstly assessed, and then stratified analyses were performed based on the types of AITD. Haplotype analysis was also conducted using Haploview 4.0 platform. P value less than 0.05 was considered statistically significant.

## RESULTS

### Demographic and clinical characteristics of subjects

Table 1 shows the demographic and clinical characteristics of all the participants in this study. In the GD group, 464 (70.2%) patients were females, 197 (29.8%) patients were males, and the mean age was 36.9 years (Table 1). The HT group consisted of 274 (80.1%) female patients and 68 male patients, and their mean age was 34.8 years (Table 1). In the control group, 587 (66.7%) individuals were females, 293 (33.3%) individuals were males, and the mean age was 38.8 years (Table 1). No significant difference concerning age and gender was observed among those three groups, and all P values were greater than 0.05 (Table 1).

**Table 1:** Demographic and clinical characteristics of all the participants in this study

Characteristics	GD (N = 661)	HT (n = 342)	Controls (N = 880)
Sex (%)			
Male	197 (29.8)	68 (19.9)	293 (33.3)
Female	464 (70.2)	274 (80.1)	587 (66.7)
Age (Mean ± SD, year)	36.9 ± 14.5	34.8 ± 13.8	38.8 ± 9.0
Onset age (Mean ± SD, year)	33.9 ± 14.5	32.4 ± 13.4	NA*
Thyroid size (%)			
Normal	116 (17.6)	48 (14.0)	880 (100.0)
First degree	109 (16.5)	56 (16.4)	0 (0.0)
Second degree	347 (52.5)	213 (62.3)	0 (0.0)
Third degree	89 (13.4)	25 (7.3)	0 (0.0)
Family history (%)			
Yes	134 (20.27)	70 (20.47)	0 (0.0)
No	527 (79.73)	272 (79.53)	880 (100.0)
Ophthalmopathy (%)			
Yes	115 (17.40)	6 (1.75)	0 (0.0)
No	546 (82.60)	336 (98.25)	880 (100.0)

* NA: not available.

### Allele and genotyping results

Genotype distributions for the loci of rs294185, rs292001, rs665691, rs294179 and rs682658 were all confirmed to HWE in both patients and controls (P > 0.05). Table 2 shows the frequencies of alleles and genotypes for those five SNPs in patients and controls.

**Table 2:** Genotype distributions and allele frequencies of C1q SNPs in AITD patients and controls

SNPs	Allele or genotypes	Controls (%)	AITD (%)	P values	GD (%)	P values	HT (%)	P values
rs294185	CC	304 (34.5)	361 (36.0)	0.72	235 (35.5)	0.78	126 (36.8)	0.75
	CT	411 (46.7)	466 (46.5)		311 (47.0)		155 (45.3)	
	TT	165 (18.7)	176 (17.5)		115 (17.4)		61 (17.8)	
	C	1019 (57.9)	1188 (59.2)	0.41	781 (59.1)	0.51	407 (59.5)	0.470
	T	741 (42.1)	818 (40.8)		541 (40.9)		277 (40.5)	
rs292001	AA	369 (41.9)	410 (40.9)	0.89	269 (40.7)	0.88	141 (41.2)	0.97
	AG	398 (45.2)	463 (46.2)		307 (46.4)		156 (45.6)	
	GG	113 (12.8)	130 (12.9)		85 (12.9)		45 (13.2)	
	A	1136 (64.5)	1283 (64.0)	0.71	845 (63.9)	0.72	438 (64.0)	0.81
	G	624 (35.5)	723 (36.0)		477 (36.1)		246 (36.0)	
rs665691	CC	368 (41.8)	409 (40.8)	0.90	269 (40.7)	0.90	140 (40.9)	0.94
	CG	399 (45.3)	463 (46.2)		307 (46. 4)		156 (45.6)	
	GG	113 (12.8)	131 (13.0)		85 (12.9)		46 (13.5)	
	C	1135 (64.5)	1281 (63.9)	0.69	845 (63.9)	0.74	436 (63.7)	0.73
	G	625 (35.5)	725 (36.1)		477 (36.1)		248 (36.3)	
rs294179	AA	110 (12.5)	134 (13.4)	0.43	87 (13.2)	0.49	47 (13.7)	0.66
	AG	405 (46.0)	432 (43.1)		284 (43.0)		148 (43.3)	
	GG	365 (41.5)	437 (43.5)		290 (43.8)		147 (43.0)	
	A	625 (35.5)	700 (34.9)	0.69	458 (34.6)	0.62	242 (35.4)	0.95
	G	1135 (64.5)	1306 (65.1)		864 (65.4)		442 (64.6)	
rs682658	GG	116 (13.2)	132 (13.2)	0.83	86 (13.0)	0.81	46 (13.5)	0.96
	GT	393 (44.7)	461 (45.9)		306 (46.3)		155 (45.3)	
	TT	371 (42.2)	410 (40.9)		269 (40.7)		141 (41.2)	
	G	625 (35.5)	725 (36.1)	0.69	478 (36.2)	0.71	247 (36.1)	0.78
	T	1135 (64.5)	1281 (63.9)		844 (63.8)		437 (63.9)	

SNP: single nucleotide polymorphism; AITD: autoimmune thyroid diseases; GD: Graves’ disease; HT: Hashimoto’s thyroiditis.

No statistically significant difference was observed between AITD patients and controls in the frequencies of alleles of rs294185 (P = 0.41), rs292001 (P = 0.71), rs682658 (P = 0.68), rs665691 (P = 0.68) and rs294179 (P = 0.69) (Table 2). There was also no statistically significant difference between AITD patients and controls in the frequencies of genotypes of rs294185 (P = 0.72), rs292001 (P = 0.89), rs682658 (P = 0.83), rs665691 (P = 0.90) and rs294179 (P = 0.43) (Table 2).

Stratified analyses showed that none of those five SNPs in *C1q* gene were associated with GD or HT (all P values > 0.05) (Table 2).

### Haplotype analysis

**Figure 1:**
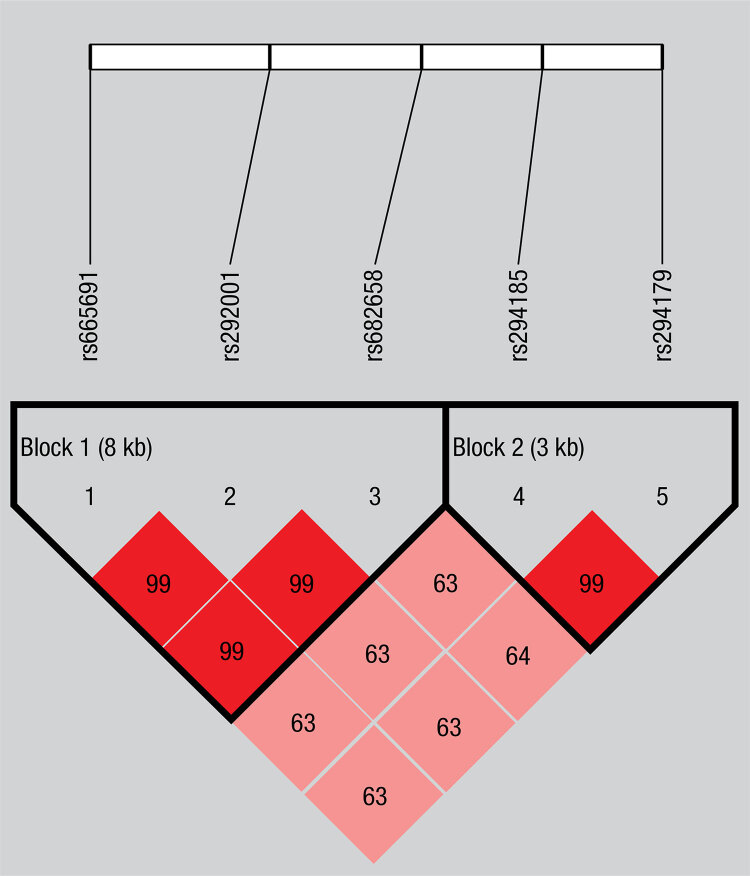
Linkage disequilibrium (LD) block defined by the Haploview 4.2.

Haplotype analysis suggested strong linkage disequilibrium (LD) in those five SNPs of *C1q* gene (Table 3). LD mainly existed between rs665691 and rs292001, rs292001 and rs682658, rs665691 and rs682658, rs294185 and rs294179. Table 4 shows the frequency of each haplotype. However, there were no obvious associations of the haplotypes of block 1 and block 2 with GD or HT (Table 4). Figure 1 shows two detected LD blocks according to D’ value, which were rs665691-rs292001-rs682658 and rs294185-rs294179, respectively.

**Table 3:** Linkage disequilibrium in those five SNPs of C1q gene

L1	L2	D’		r^2^	

		Control	AITD	Control	AITD
rs665691	rs292001	1.0	0.998	0.998	0.991
rs292001	rs682658	0.998	0.993	0.973	0.983
rs665691	rs682658	0.988	0.991	0.975	0.983
rs294185	rs294179	0.997	0.992	0.753	0.767

**Table 4:** Haplotype analysis in AITD patients and controls

Haplotypes	Control (N, %)	AITD (N, %)	P values	GD (N, %)	P values	HT (N, %)	P values
CAT	1129 (64.2)	1273 (63.7)	0.74	844 (63.8)	0.83	429 (63.5)	0.73
GGG	619 (35.2)	719 (36.0)	0.62	477 (36.1)	0.62	242 (35.8)	0.78
CG	1018 (57.8)	1185 (59.1)	0.44	779 (58.9)	0.54	405 (59.4)	0.49
TA	624 (35.5)	697 (34.7)	0.65	456 (34.5)	0.58	241 (35.3)	0.94
TG	117 (6.6)	121 (6.0)	0.44	85 (6.4)	0.81	36 (5.3)	0.21

There were five tag SNPs on the LD map of C1q gene. Patterns of LD between the C1q gene SNPs were shown. The strength of LD measured by D’ multiplying 100 was displayed in the red diamonds. A larger number in the cell indicates a higher degree of LD.

## DISCUSSION

Polymorphisms in the complement *C1q* gene have been reported to be associated with several types of autoimmune diseases. However, their roles in AITD still remain unclear. In the present study, we aimed to assess the associations of *C1q* gene polymorphisms with AITD susceptibility. To the best of our knowledge, we, for the first time, clarified the roles of *C1q* gene SNPs in AITD susceptibility. We recruited a set of 1,003 AITD patients (661 with GD and 342 with HT) and 880 ethnically- and geographically-matched controls from Chinese Han population, and examined the associations of five commonly detected SNPs in *C1q* gene with AITD susceptibility. In the present study, we showed that AITD patients and healthy controls had statistically similar frequencies of genotypes and alleles of rs294185, rs292001, rs682658, rs665691 and rs294179 (Table 2). Haplotype analysis, which can provide powerful and conducive analyses in identifying genes associated with complex diseases (17), also did not find obvious associations of *C1q* gene with AITD susceptibility. Therefore, our findings suggested that those five common SNPs in *C1q* gene (rs294185, rs292001, rs682658, rs665691 and rs294179) were not associated with AITD susceptibility in Chinese Han population.

The complement system is a vital part of immune system in human body, and it is involved in both innate and adaptive immune systems. As an important part of C1, C1q plays important roles in the clearance of apoptotic cells and immune complexes (18). C1q deficiency is the first identified single-gene defect, which causes lupus-like disease (19). Patients with C1q deficiency can develop lupus with high penetrance (18). It has been reported that more than 90% of individuals with complete congenital deficiency of C1q can develop early-onset photosensitive SLE (14). The presence of anti-C1q has been also strongly correlated with hypocomplementemia, disease activity and renal involvement in SLE patients (20). Several SNPs have been also found in the *C1q* gene, such as rs292001, rs682658, rs665691 and rs294179. It has been reported that the A allele and AA genotype of *C1q* rs292001 can be considered a risk factor for juvenile SLE and lupus nephritis in a cohort of Egyptian children (21).

Goulielmos and cols. reported that *C1q* rs292001 is associated with type 2 diabetes mellitus (22). Other genetic polymorphisms of *C1q* have been also associated with susceptibility to autoimmune diseases, such as rheumatoid arthritis (23). Since C1q deficiency can result in increased susceptibility to lupus-like autoimmune disease, the genetic polymorphisms of *C1q* may also have important roles in SLE (24). In addition, genetic deficiencies of *C1q* in mice can also lead to autoimmunity (25,26). The above-mentioned findings suggest that C1q is intensively involved in autoimmunity, and genetic polymorphisms in *C1q* are possibly associated with some types of autoimmune diseases.

It has been known that many factors are involved in the initiation and development of AITD, such as genetic factors, environmental factors and nutritional factors. However, the exact pathogenesis of AITD still remains poorly explored. Previous studies have suggested that some genetic factors are intensively involved in the initiation of immune responses against the thyroid gland during the development of AITD, such as *HLA* and *CTLA4* (2). AITD is characterized by autoimmunity, and C1q has been suggested to have some roles in AITD. Potlukova and cols. reported that auto-antibodies against C1q are more prevalent in AITD patients compared with controls (27), while Brohee and cols. reported that circulating immune complexes containing C1q are also more prevalent in AITD patients than controls (28). All of the above evidence suggests that *C1q* gene is probably an important element associated with AITD. However, our data failed to identify obvious associations of those five different SNPs with AITD susceptibility. Therefore, it is necessary to explore the roles of *C1q* gene in AITD in future studies.

There were several limitations in our study. First, the findings from our study were not sufficient to explore the full roles of *C1q* gene in AITD, since we only explored five common SNPs in the *C1q* gene. Future studies can further investigate the associations of other SNPs in the *C1q* gene with AITD susceptibility. In addition, our study was carried out in only Chinese Han population, which could not be generalized to other ethnical populations. The roles of *C1q* gene polymorphisms in AITD susceptibility in Caucasian or African populations need to be studied in future studies. Second, the sample size in our study might not be enough to detect a modest association of *C1q* gene with AITD susceptibility. More studies with larger sample size are still required to further identify those SNPs carrying a smaller risk effect. Finally, we did not analyze the gene-environment interaction in our study due to the limitation of study design. Prospective studies in the future may explore the possible gene-environment interaction in the associations of *C1q* gene polymorphisms with AITD susceptibility. In conclusion, our findings suggested that five common SNPs in *C1q* gene (rs294185, rs292001, rs682658, rs665691 and rs294179) were all not associated with AITD susceptibility in Chinese Han population. Future studies are required to investigate the associations of those *C1q* gene SNPs with AITD susceptibility in Caucasian or African populations. In addition, it is also necessary to explore the associations of other SNPs in *C1q* gene with AITD susceptibility in future studies.
